# Examination of the genetic basis for sexual dimorphism in the *Aedes aegypti* (dengue vector mosquito) pupal brain

**DOI:** 10.1186/s13293-014-0010-x

**Published:** 2014-10-21

**Authors:** Michael Tomchaney, Keshava Mysore, Longhua Sun, Ping Li, Scott J Emrich, David W Severson, Molly Duman-Scheel

**Affiliations:** 1 Eck Institute for Global Health, Galvin Life Sciences, University of Notre Dame, Notre Dame 46556, IN, USA; 2 Department of Biological Sciences, Galvin Life Sciences, University of Notre Dame, Notre Dame 46556, IN, USA; 3 Department of Medical and Molecular Genetics, Indiana University School of Medicine, Raclin-Carmichael Hall, South Bend 46617, IN, USA; 4 Department of Computer Science and Engineering, University of Notre Dame, Notre Dame 46556, IN, USA

**Keywords:** Aedes aegypti, Mosquito, Vector, Pupae, Brain, Nervous system, Dimorphism, Doublesex, Development, Optic lobe

## Abstract

**Background:**

Most animal species exhibit sexually dimorphic behaviors, many of which are linked to reproduction. A number of these behaviors, including blood feeding in female mosquitoes, contribute to the global spread of vector-borne illnesses. However, knowledge concerning the genetic basis of sexually dimorphic traits is limited in any organism, including mosquitoes, especially with respect to differences in the developing nervous system.

**Methods:**

Custom microarrays were used to examine global differences in female vs. male gene expression in the developing pupal head of the dengue vector mosquito, *Aedes aegypti.* The spatial expression patterns of a subset of differentially expressed transcripts were examined in the developing female vs. male pupal brain through *in situ* hybridization experiments. Small interfering RNA (siRNA)-mediated knockdown studies were used to assess the putative role of Doublesex, a terminal component of the sex determination pathway, in the regulation of sex-specific gene expression observed in the developing pupal brain.

**Results:**

Transcripts (2,527), many of which were linked to proteolysis, the proteasome, metabolism, catabolic, and biosynthetic processes, ion transport, cell growth, and proliferation, were found to be differentially expressed in *A. aegypti* female vs. male pupal heads. Analysis of the spatial expression patterns for a subset of dimorphically expressed genes in the pupal brain validated the data set and also facilitated the identification of brain regions with dimorphic gene expression. In many cases, dimorphic gene expression localized to the optic lobe. Sex-specific differences in gene expression were also detected in the antennal lobe and mushroom body. siRNA-mediated gene targeting experiments demonstrated that Doublesex, a transcription factor with consensus binding sites located adjacent to many dimorphically expressed transcripts that function in neural development, is required for regulation of sex-specific gene expression in the developing *A. aegypti* brain.

**Conclusions:**

These studies revealed sex-specific gene expression profiles in the developing *A. aegypti* pupal head and identified Doublesex as a key regulator of sexually dimorphic gene expression during mosquito neural development.

## 1 Background

Vector mosquitoes inflict more human suffering than any other organism*—*and kill more than one million people each year. *Aedes aegypti* is the primary vector for dengue, the most widespread and significant arboviral disease in the world. Dengue is presently a threat to >2.5 billion people in the tropics with a yearly incidence of approximately 50 million cases resulting in approximately 22,000 deaths annually worldwide. The *A. aegypti* genome project [1] greatly facilitated efforts to study the biology of this mosquito, but the genetic regulation of mosquito developmental biology is still poorly understood. Our laboratory has begun to address this need by pursuing functional developmental genetic studies in *A. aegypti*[2]-[8]. Here, we examine the genetic basis of sexual dimorphism during *A. aegypti* development.

Most animal species exhibit sexually dimorphic behaviors, many of which are linked to sexual reproduction [9]. Mosquitoes, which display innate sexually dimorphic behaviors that contribute to the spread of human disease, are excellent subjects for studies that explore the biological basis of sexual dimorphism. For example, only female adult mosquitoes, which require a blood meal for reproduction, bite humans and transmit disease. If identified, the genes which regulate the development of adult female blood feeding behavior could represent novel targets for vector control. To date, the analysis of mosquito sexual dimorphism has primarily focused on understanding differences between adult female and male mosquitoes, particularly with respect to sex-specific behaviors related to disease transmission (for example [10]). However, sexually dimorphic phenotypes, including behaviors, are the products of differential gene expression that initiates during development and therefore must also be studied during development. We presently lack knowledge concerning which genes regulate the development of sexually dimorphic traits in mosquitoes.

Our extremely limited knowledge of the developmental genetic basis for sexual dimorphism in insects is largely restricted to *Drosophila melanogaster*, a genetically tractable*—*albeit highly derived*—*dipteran insect that exhibits innate sexually dimorphic behaviors. The genetic tractability of the *Drosophila* system has facilitated analysis of the developmental genetic basis for sexual dimorphism in this species. The *Drosophila doublesex* (*dsx*) gene encodes a terminal transcription factor in the sex determination pathway (reviewed by [11],[12]). The pre-mRNAs of *dsx* are sex-specifically spliced [13],[14], giving rise to male (DsxM) and female (DsxF) proteins. Male and female Dsx splice variants have a common DNA-binding domain, but distinct C termini that differentially direct sex-specific gene expression (reviewed by [11],[12]). Although research has begun to elucidate the genetic basis of sexually dimorphic development in *Drosophila*, more work is necessary not only in *Drosophila*, but also in non-model insects, including disease vector mosquitoes. Moreover, our detection of numerous differences between mosquito and *Drosophila* development underscores the importance of analyzing *dsx* gene function directly in mosquito species of interest [3]-[5],[15].

Male and female splice variants of the *dsx* gene have been detected in *A. aegypti*[16]. Although these sex-specific *dsx* splice forms are believed to differentially regulate target gene transcription and sex-specific development in males and females, this hypothesis has yet to be functionally tested in mosquitoes. Unfortunately, exploration of the roles of *dsx* and the developmental genetic basis for sexual dimorphism in mosquitoes has been hampered by a lack of methodology to pursue functional genetic studies during mosquito development. Here, we examine differences in gene expression in the developing *A. aegypti* pupal brain. We then use small interfering RNA (siRNA)-mediated gene targeting to demonstrate that Dsx regulates dimorphic gene expression in the developing *A. aegypti* central nervous system.

## 2 Methods

### 2.1 Animal rearing

The *A. aegypti* Liverpool-IB12 (LVP-IB12) strain, from which the current genome sequence [1] was generated, was used in these studies. These mosquitoes were reared as described [17], except that an artificial membrane blood feeding system was employed. Mosquitoes were maintained in an insectary at 26°C, approximately 80% humidity, under a 12-h light and 12-h dark cycle with 1-h crepuscular periods at the beginning and end of each light cycle. Mosquito larvae were fed a suspension of dried beef liver powder, while adults were provided cotton soaked with 10% sugar solution.

### 2.2 Microarray experiments

Tissues for the microarray studies were prepared as follows: age-matched (±15 min) male and female pupae were raised to 24 h after puparium formation (APF) and sexed on the basis of differing tail morphology as previously described [18]. Although our own primary interest is the pupal brain, dimorphic gene expression was assessed in male vs. female head tissue since removal of the head (as opposed to the more time-consuming dissection of the brain) can be performed quickly and promotes minimization of temporal changes in gene expression between males and females or among replicate experiments. Twenty male and female heads were microdissected for each of four replicates during daylight hours at roughly the same time of day (approximately 11 a.m.) so that circadian-related changes in gene expression would not be a confounding factor. Following dissection, head tissue was immediately stored in RNAlater (Ambion, Austin, TX, USA).

For each of four replicates, RNA extraction was performed with the RNeasy Mini Kit (Qiagen, Valencia, CA, USA) according to the manufacturer's instructions. Total RNA was quantified spectrophotometrically, and RNA quality was assessed using an Agilent 2100 Bioanalyzer (Santa Clara, CA, USA). Both the quantity and quality of the RNA preps were deemed to be suitable. RNA samples were then submitted to the Notre Dame Genomics and Bioinformatics Core Facility (Notre Dame, IN, USA), which performed hybridization experiments using the *A. aegypti* 12-plex microarray design: 090305_Aedes_aegypti_TEfam_expr.ndf (Gene Expression Omnibus platform GPL18530) developed by Susanta K. Behura and David W. Severson in conjunction with Roche NimbleGen. On this microarray, 16,581 genes/ORFs are represented. Each gene/ORF is represented by 1 to 3 unique 60-mer probe sequences that are synthesized in triplicate on the microarray (effectively 3 to 9 probes per gene). RNA labeling and hybridization experiments were performed according to the manufacturer's instructions. Four unique replicates (male 1, 2, 3, 4 and female 1, 2, 3, 4) and two repeat replicates (male 5, which is a repeat hybridization of male 1; male 6, a repeat hybridization of male 4; female 5, a repeat hybridization of female 1; female 6, a repeat hybridization of female 2) were assessed in the hybridization experiment. Microarrays were scanned using a NimbleGen MS 200 Microarray Scanner (Roche NimbleGen, Madison, WI, USA). Data were extracted and analyzed using NimbleScan Version 2.5 software (Roche NimbleGen). Microarray data pre-processing and normalization were performed using the Bioconductor packages in R Version 3.0.1. Raw gene expression data were log2-transformed and normalized using the quantile normalization method [19]. The Student's *t* test was used for analysis of statistical significance. Raw *p* values were adjusted using the Bonferroni procedure for control of false discovery rate. Microarray data were evaluated with DAVID [20] and GenMAPP2 [21] to identify GO terms and KEGG pathways (Koyota Encyclopedia of Genes and Genomes). Data were deposited at Gene Expression Omnibus (accession number GSE56521).

### 2.3 Whole mount *in situ* hybridization

*In situ* hybridization was performed as described [22] on male vs. female pupal brain tissue prepared as discussed in [6],[8],[23]. Riboprobes were synthesized per the Patel [24] protocol to the following genes: *arrow* (*VB:AAEL009806*), *caspase 7* (*VB:AAEL012143*), *cyclin-dependent kinase 4/6* (*cdk4/6; VB:AAEL001407*), *cubitus interruptus* (*ci; VB:AAEL012039*), *dsx* (*VB:AAEL009114*), *geko* (*VB:AAEL006310*), *odorant binding protein 10* (*obp10; VB:AAEL007603*), *odorant binding protein 13* (*obp13; VB:AAEL002591*), *p53* (*VB:AAEL007594*), *rab6* (*VB:AAEL006091*), *synaptojanin* (*synj; VB:AAEL011417*), and *takeout* (*VB:AAEL011966*)*.* These gene numbers correspond to Vectorbase (VB) [25], which was also the source for all orthology assignments in this study. Based on information provided in Salvemini et al. [16], probes corresponding to the following *dsx* exons were synthesized: (i) exon 2, which is common to both the male and female splice forms, (ii) exon 5a, which is female-specific, and (iii) exons 4 combined with 6, which are spliced together in males, but separated by exons 5a/b in females.

At least 25 tissue specimens from each sex were used in each *in situ* hybridization reaction, and experiments were replicated minimally three times. In addition to confirming dimorphic gene expression at 24 h APF, which corresponds to the time in which the pupal brains were gathered for microarray analyses, dimorphic gene expression was also verified at both 22 and 26 h APF. This allowed for confirmation that the dimorphic expression observed was not simply a result of slight age differences between male and female pupae. As discussed above, such differences were experimentally minimized through use of narrow 30-min animal collection windows. Sense riboprobes served as controls in all hybridization experiments, which routinely yielded unlabeled brains. Following processing, colorimetrically stained tissues were mounted and analyzed using a Zeiss Axioimager equipped with a Spot Flex camera (Oberkochen*,* Germany). Images were processed with Adobe Photoshop software. Double labeling experiments were performed with gene-specific probes in conjunction with anti-HRP (Jackson Immunoresearch, West Grove, PA, USA) detected with goat anti-rabbit FITC (Jackson Immunoresearch) as described [22]. Imaging of these specimens was performed with a Zeiss 710 confocal microscope using Zen software, and scanned images were analyzed using FIJI and Adobe Photoshop software.

For *in situ* hybridization experiments on sectioned brain tissues, paraffin embedding and sectioning was performed generally as described in [26]. Blocks were mounted and sectioned at 12 μm thickness on a Leica RM2155 Rotary Microtome (Leica Microsystems GmbH, Nussloch, Germany). Following deparaffinization, which was performed as described in the Abcam IHC-Paraffin Protocol (IHC-P), slides were placed in LockMailer Microscope Slide Jars (Simport Scientific, Beloeil, QC, USA), rehydrated, and then processed as described above.

### 2.4 Immunohistochemical staining

Immunohistochemical staining was performed as described earlier [6],[23],[27]. Rat anti-DN-cadherin developed by T. Uemura was obtained from the Developmental Studies Hybridoma Bank, which was created by the NICHD-NIH and is maintained at the University of Iowa, and was used at a concentration of 1:50 to visualize neuropile domains in the *A. aegypti* brain. Alexa Fluor 568 goat anti-rat IgG secondary antibody (Life Technologies) was used at a concentration of 1:200. Nuclear counterstaining was performed with TOTO-3 iodide (Molecular Probes, Grand Island, NY, USA). Double labeling immunohistochemical experiments combined with *in situ* hybridization were performed with gene-specific probes in conjunction with anti-HRP (Jackson Immunoresearch) detected with goat anti-rabbit FITC (Jackson Immunoresearch) as described in Haugen et al. [22]. Imaging of fluorescently stained specimens was performed with a Zeiss 710 confocal microscope using Zen software, and scanned images were analyzed using FIJI and Adobe Photoshop software.

### 2.5 Analysis of Dsx consensus binding sites

*A. aegypti* scaffold locations that differ by no more than one mismatch to the Dsx binding site consensus GCAACAATGTTGC [28] were identified using a custom Perl script, which is available at https://bitbucket.org/NDBL/bindingsearch/. First, the consensus and all 13-mers that differ at a single base (3 alternative bases × 13 total positions = 39 alternative 13-mers) were placed into a hash data structure. Next, each 13-mer in the *A. aegypti* genome was checked against the hash to quickly determine valid locations. Genes in which the consensus sites resided, as well as open reading frames located within 10,000 kB upstream or downstream of the consensus sites, were identified using the gene browser tool in Vectorbase [25]. GO terms associated with these genes were identified with DAVID [20].

### 2.6 RNAi knockdown experiments

Knockdown of *dsx* was performed with two different siRNA duplexes, *dsx KD A* and *dsx KD B*, both of which were designed to target the male and female *dsx* splice forms. The two siRNAs correspond to different target sequences in exon 2, which is common to both the male and female *dsx* splice variants. The sequences of the siRNA duplexes are as follows: *dsx KD A:* 5′AGAGAUGAUCCAUAAUUCUCAGCAG3′/5′UUUCUCUACUAGGUAUUAAGAGUCGUC3′ and *dsx KD B:* 5′CAGGAACAGACGACGAACUUGUCAA3′/5′GAGUCCUUGUCUGCUGCUUGAACAGUU3′. These siRNAs were designed using Integrated DNA Technology (IDT) software and chemically synthesized by IDT. The siRNAs were confirmed through Blast searches to have no significant homology to *A. aegypti* genes other than *dsx.* All phenotypes were confirmed following knockdown with both *dsx KD A and dsx KD B*, suggesting that none of the phenotypes reported here were the result of off-site targeting by either siRNA. A scrambled version of *dsx KD B*, an siRNA duplex lacking significant sequence homology to any genes in the *A. aegypti* genome, was used in control experiments: 5′ACAGUACCAGUGGACACAUACGG 3′/5′AUAGCCACAGACAUGAGCGGCAU3′. None of the phenotypes reported were observed in control-injected animals, which resembled wild-type animals.

siRNAs were delivered using a modification of our embryo microinjection protocol [29] and the Blitzer et al. [30] larval injection protocol. In summary, age-synchronized freshly emerged pupae were anesthetized in ice water for 5 min prior to injection and placed on dry filter paper. Animals were injected with control or KD siRNAs vertical to the body axis in the thoracic region. Following injection, pupae were placed in water and maintained through 24 h APF. Fifty individuals were injected per treatment for each replicate, and experiments to confirm each phenotype reported here were repeated in triplicate. Knockdown was verified through *in situ* hybridization according to the protocol described above and through use of riboprobes corresponding to the common, male-specific, and female-specific splice forms (see above).

## 3 Results

### 3.1 Detection of global gene expression differences in the pupal head

**Figure 1:**
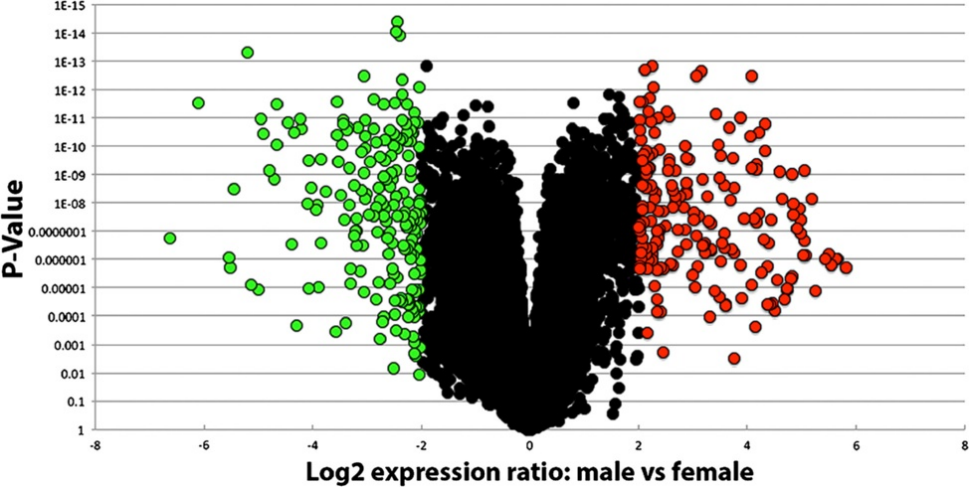
**Sex-biased gene expression in the pupal head.** A volcano plot comparing significance vs. fold change for male vs. female 24 h pupal head transcriptome data is shown. The red dots denote 189 genes that were found to be significantly upregulated in males (*p* < 0.05) with a log2 fold change greater than 2. Green dots represent the 201 genes that were found to be significantly upregulated in females (*p* < 0.05) with a log2 fold change greater than 2. Black dots represent genes with a log2 fold change less than 2.

A cDNA microarray experiment was conducted to profile and compare pupal head transcript expression in female vs. male *A. aegypti* pupae. Head tissues from 24 h pupae were selected for these studies, as our recent work has identified this time point, which follows pupal histolysis, to be a critical period for nervous system development [6],[8],[23]. Microarray analysis of 16,581 genes/ORFs revealed differential gene expression among a number of transcripts in the male vs. female pupal head transcriptome. The dimorphism microarray experiment uncovered 2,527 significant differentially expressed transcripts (DETs) (Additional file 1). A volcano plot comparing significance vs. fold change for these data is displayed in Figure 1. One hundred eighty-nine DETs were found to be significantly upregulated in males (*p* < 0.05) with a log2 fold change greater than 2.0, while 201 DETs were significantly upregulated in females (*p* < 0.05) with a log2 fold change greater than 2.0.

**Figure 2:**
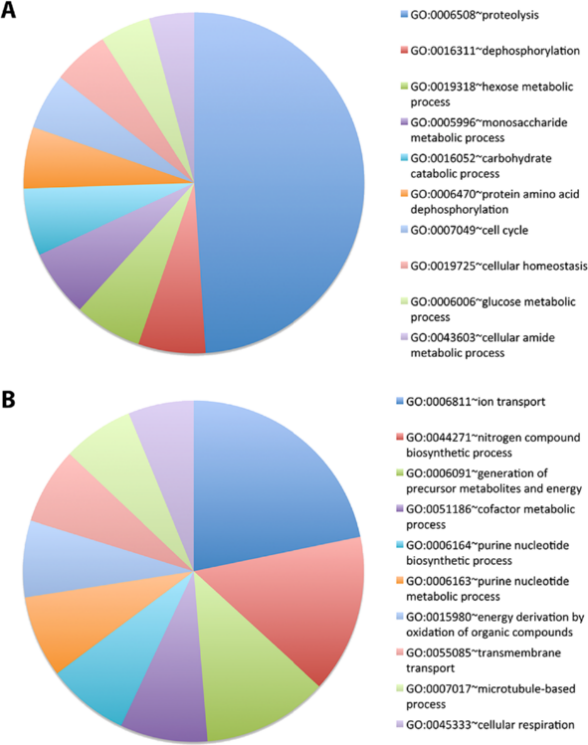
**Over-represented GO categories among sex-biased genes expressed in the pupal head.** The top ten significant GO terms (*p* < 0.05) associated with genes that were significantly (*p* < 0.05) upregulated in female **(A)** or male **(B)** 24 h pupal heads are indicated.

**Figure 3:**
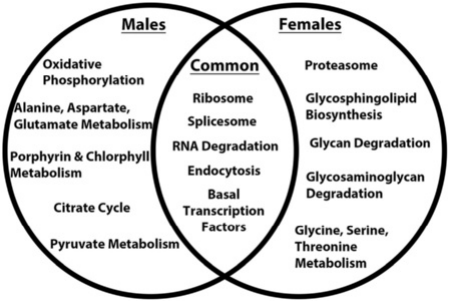
**Differential distribution of KEGG pathway genes in male vs. female pupal heads.** Pathways in which the number of significant DETs is significantly (*p* < 0.05) higher in male or female 24 h pupal heads are indicated. A number of pathways were commonly activated in females and males without statistically significant bias (common pathways). An itemized list of genes corresponding to each pathway is provided in Additional file 3.

Microarray data were evaluated with DAVID [20] and GenMAPP [21] to identify gene ontology terms and KEGG pathways significantly associated with sex-specific DETs. Significantly upregulated DETs in the female pupal head are primarily linked to the proteolytic, metabolic, catabolic, protein dephosphorylation, homeostatic, cell cycle, and microtubule-based movement GO terms (Figure 2A, Table 1). A large number of the DETs significantly upregulated in females are related to proteolysis (Figure 2A), with many of the transcripts encoding serine proteases (Table 2). KEGG pathway analysis of DETs significantly upregulated in female pupal heads identified the proteasome, glycosphingolipid biosynthesis, glycosaminoglycan, and other glycan degradation, as well as glycine/serine/threonine metabolism as significant pathways (Figure 3).

**Table 1:** Significantly over-represented GO categories associated with DETs upregulated in female pupal heads

**Term**	**Count**	***p*** **value**
GO:0006508—proteolysis	92	3.17E − 06
GO:0016311—dephosphorylation	12	0.002031467
GO:0019318—hexose metabolic process	12	0.009445235
GO:0005996—monosaccharide metabolic process	12	0.013314328
GO:0016052—carbohydrate catabolic process	12	0.020208459
GO:0006470—protein amino acid dephosphorylation	11	0.001514357
GO:0007049—cell cycle	10	0.002262897
GO:0019725—cellular homeostasis	10	0.033082
GO:0006006—glucose metabolic process	9	0.025805435
GO:0043603—cellular amide metabolic process	8	0.00250685
GO:0006733—oxidoreduction coenzyme metabolic process	8	0.009081419
GO:0045454—cell redox homeostasis	8	0.039387289
GO:0006098—pentose-phosphate shunt	7	6.34E − 04
GO:0006739—NADP metabolic process	7	6.34E − 04
GO:0019748—secondary metabolic process	7	0.002617106
GO:0006769—nicotinamide metabolic process	7	0.002617106
GO:0009820—alkaloid metabolic process	7	0.002617106
GO:0046496—nicotinamide nucleotide metabolic process	7	0.002617106
GO:0019362—pyridine nucleotide metabolic process	7	0.007431131
GO:0006007—glucose catabolic process	7	0.047566184
GO:0019320—hexose catabolic process	7	0.047566184
GO:0006913—nucleocytoplasmic transport	6	0.0364855
GO:0051169—nuclear transport	6	0.0364855
GO:0046700—heterocycle catabolic process	5	0.026037743
GO:000716—enzyme linked receptor protein signaling pathway	5	0.026037743
GO:0009070—serine family amino acid biosynthetic process	4	0.008178934
GO:0006564—L-serine biosynthetic process	3	0.017716355
GO:0042176—regulation of protein catabolic process	3	0.017716355
GO:0009894—regulation of catabolic process	3	0.017716355
GO:0019439—aromatic compound catabolic process	3	0.017716355

Over-represented GO categories among sex-biased genes expressed in the female 24 h pupal head are shown. The number of DETs associated with each GO term (count) and corresponding Fisher's exact test *p* values are indicated.

**Table 2:** Genes upregulated in the female pupal head are associated with proteolysis

**Gene**	**Description**	**Expression**
AAEL000028	CLIPB34 Clip-domain serine protease family B	2.5
AAEL018109	Hypothetical protein	6.0
AAEL000037	CLIPB35 Clip-domain serine protease family B	4.0
AAEL000038	CLIPB6 Clip-domain serine protease family B	4.3
AAEL000074	CLIPB1 Clip-domain serine protease family B	4.0
AAEL000224	Serine protease	2.2
AAEL001077	CLIPB45 Clip-domain serine protease family B	8.8
AAEL001084	CLIPB21 Clip-domain serine protease family B	9.4
AAEL001098	Clip-domain serine protease	3.7
AAEL002124	CLIPD6 Clip-domain serine protease family D	11.2
AAEL002126	CLIPA15 Clip-domain serine protease family A	5.3
AAEL002585	Serine protease	2.1
AAEL002590	Serine protease	2.7
AAEL002593	Serine protease	3.0
AAEL002595	Serine protease	5.0
AAEL002600	Serine protease	2.0
AAEL002629	Serine protease	5.1
AAEL002767	Conserved hypothetical protein	5.1
AAEL003243	CLIPB13A Clip-domain serine protease family B	3.1
AAEL003251	Serine protease snake	2.7
AAEL003276	Hypothetical protein	3.3
AAEL003280	CLIPB26 Clip-domain serine protease family B	3.8
AAEL003610	CLIPB9 Clip-domain serine protease family B	2.3
AAEL004518	CLIPC5A Clip-domain serine protease family C	6.0
AAEL004524	CLIPC5B Clip-domain serine protease family C	8.2
AAEL004540	CLIPC6 Clip-domain serine protease family C	3.1
AAEL004979	CLIPD2 Clip-domain serine protease family D	2.1
AAEL005064	CLIPB5 Clip-domain serine protease family B	4.4
AAEL006136	Serine protease	3.0
AAEL006151	Serine protease	3.7
AAEL006434	Serine protease	4.5
AAEL006708	Hedgehog	2.3
AAEL007107	Serine protease	2.3
AAEL007511	Serine protease	8.5
AAEL007597	CLIPC3 Clip-domain serine protease family C	2.8
AAEL007602	Trypsin	3.5
AAEL007969	Serine protease	4.5
AAEL010267	Serine protease	3.4
AAEL010270	Hypothetical protein	9.1
AAEL010780	Carboxypeptidase	3.6
AAEL010866	Serine protease	4.0
AAEL010867	Serine protease	10.8
AAEL011324	Hypothetical protein	3.0
AAEL011991	CLIPC1 Clip-domain serine protease family C	2.0
AAEL012143	CaspaseS7	3.3
AAEL012500	Ubiquitin-protein ligase	2.2
AAEL012713	CLIPC16 Clip-domain serine protease family C	2.9
AAEL012775	Serine protease	3.3
AAEL012777	Serine protease snake	3.5
AAEL012780	Trypsin	7.4
AAEL012785	CLIPB23 Clip-domain serine protease family B	4.1
AAEL012797	Serine protease	3.4
AAEL013245	CLIPB28 Clip-domain serine protease family B	10.1
AAEL013299	Serine protease	2.6
AAEL014344	Adam (a disintegrin and metalloprotease)	5.3
AAEL014567	Oviductin	10.1
AAEL015439	CLIPD7 Clip-domain serine protease family D	2.7
AAEL015527	Hypothetical protein	2.4
AAEL015533	Conserved hypothetical protein	4.1

KEGG pathway analyses indicated that proteolysis is the most represented pathway corresponding to DETs upregulated in the female pupal head. The gene numbers, descriptions, and log2 fold change with respect to males is indicated for each gene corresponding to proteolysis.

Significant DETs upregulated in male pupal heads are significantly linked to ion transport, biosynthetic, and metabolic GO terms (Figure 2B, Table 3). A majority of the DETs associated with ion transport (Figure 2B), the GO term linked to the largest number of male-specific upregulated transcripts, encode ion channels (Table 4). Pathway analysis of DETs significantly upregulated in male pupal heads identified oxidative phosphorylation, alanine/aspartate/glutamate metabolism, porphyrin and chlorophyll metabolism, the citric acid cycle, and pyruvate metabolism as significant pathways (Figure 3). Non-differentially expressed head transcripts common to both the male and female brain grouped under a number of significant pathways that were generally associated with the regulation of gene and protein expression (Figure 3).

**Table 3:** Significantly over-represented GO categories associated with DETs upregulated in male pupal heads

**Term**	**Count**	***p*** **value**
GO:0006811—ion transport	42	0.010896197
GO:0044271—nitrogen compound biosynthetic process	29	0.007748843
GO:0006091—generation of precursor metabolites and energy	23	2.70E − 04
GO:0051186—cofactor metabolic process	16	0.033341803
GO:0006164—purine nucleotide biosynthetic process	15	0.021253849
GO:0006163—purine nucleotide metabolic process	15	0.023426935
GO:0015980—energy derivation by oxidation of organic compounds	14	1.86E − 04
GO:0055085—transmembrane transport	14	0.011896142
GO:0007017—microtubule-based process	13	0.01594331
GO:0045333—cellular respiration	12	5.71E − 04
GO:0018130—heterocycle biosynthetic process	12	0.001152569
GO:0006119—oxidative phosphorylation	12	0.004475745
GO:0046394—carboxylic acid biosynthetic process	11	0.040301387
GO:0016053—organic acid biosynthetic process	11	0.040301387
GO:0007005—mitochondrion organization	9	8.07E − 07
GO:0022900—electron transport chain	9	0.003534463
GO:0016044—membrane organization	8	0.001738542
GO:0042773—ATP synthesis coupled electron transport	7	0.001700687
GO:0022904—respiratory electron transport chain	7	0.004925969
GO:0046942—carboxylic acid transport	7	0.017801566
GO:0015849—organic acid transport	7	0.017801566
GO:0009187—cyclic nucleotide metabolic process	7	0.026556712
GO:0009190—cyclic nucleotide biosynthetic process	7	0.026556712
GO:0070585—protein localization in mitochondrion	6	1.36E − 04
GO:0006626—protein targeting to mitochondrion	6	1.36E − 04
GO:0006839—mitochondrial transport	6	6.95E − 04
GO:0046068—cGMP metabolic process	6	0.005142832
GO:0006182—cGMP biosynthetic process	6	0.005142832
GO:0009064—glutamine family amino acid metabolic process	6	0.028832748
GO:0007006—mitochondrial membrane organization	5	3.74E − 04
GO:0007007—inner mitochondrial membrane organization	5	3.74E − 04
GO:0045039—protein import into mitochondrial inner membrane	5	3.74E-04
GO:0065002—intracellular protein transmembrane transport	5	0.002241034
GO:0042775—mitochondrial ATP synthesis coupled electron transport	5	0.010659342
GO:0006612—protein targeting to membrane	5	0.037429854
GO:0009084—glutamine family amino acid biosynthetic process	5	0.037429854
GO:0006561—proline biosynthetic process	4	0.03331591
GO:0033014—tetrapyrrole biosynthetic process	4	0.046519627
GO:0006560—proline metabolic process	4	0.046519627
GO:0006493—protein amino acid O-linked glycosylation	3	0.025391936
GO:0006122—mitochondrial electron transport	3	0.047602115

Over-represented GO categories among sex-biased genes expressed in the male 24 h pupal head are shown. The number of DETs associated with each GO term (count) and corresponding Fisher's exact test *p* values are indicated.

**Table 4:** Genes upregulated in male pupal heads are associated with ion transport

**Gene**	**Description**	**Expression**
AAEL001123	Hypothetical protein	10.6
AAEL001198	Sodium/solute symporter	2.3
AAEL001646	Kir3 inward-rectifying potassium channel	2.1
AAEL002299	High affinity copper transporter	2.1
AAEL004664	Hypothetical protein	2.0
AAEL004919	Hypothetical protein	3.5
AAEL005014	Transient receptor potential channel	2.0
AAEL007770	Voltage and ligand gated potassium channel	2.3
AAEL008338	Ion channel NompC	2.5
AAEL009813	Glutamate receptor 7	2.0
AAEL009856	Sodium/dicarboxylate cotransporter	3.0
AAEL011109	Hypothetical protein	3.3
AAEL011679	Ion channel NompC	2.7
AAEL015091	Hypothetical protein	3.3

KEGG pathway analyses indicated that ion transport is the most represented pathway corresponding to DETs upregulated in male pupal heads. The gene numbers, descriptions, and log2 fold change with respect to females is indicated for each ion transport-related gene.

### 3.2 Examination of the expression of DETs in the pupal brain

**Figure 4:**
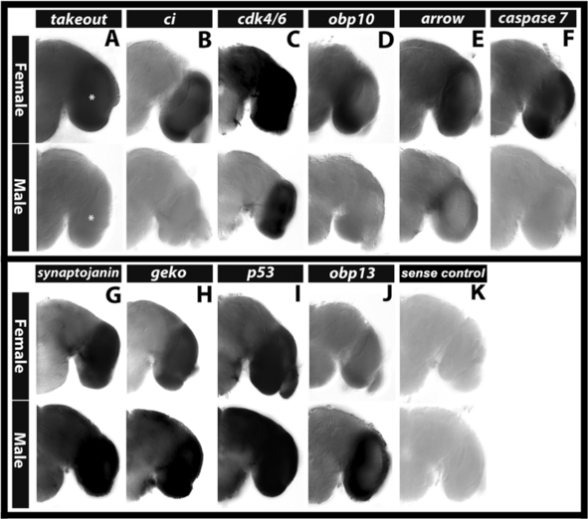
**Sex-specific expression of DETs in the*A. aegypti*pupal brain.** Differential expression of the indicated genes was confirmed in female vs. male 24 h pupal brains through whole mount *in situ* hybridization experiments. In agreement with the whole head microarray experimental data, these experiments demonstrated that *takeout*, *ci*, *cdk4/6*, *obp10*, *arrow*, and *caspase7* (top row, **A**-**F** respectively) are upregulated in the female 24 h pupal brain, while *synj*, *geko*, *p53*, and *obp13* are upregulated in the male 24 h pupal brain (**G**-**J**, respectively). A brain processed with a sense control probe lacks staining **(K)**. For a number of the genes assayed (*takeout*, *ci*, *obp10*, *arrow*, *caspase 7*, and *obp13*), dimorphic gene expression localized to the optic lobe, which is marked by an asterisk in **(A)** and located at a similar position in other panels. Brain hemisegments are oriented dorsal upward in each panel.

**Figure 5:**
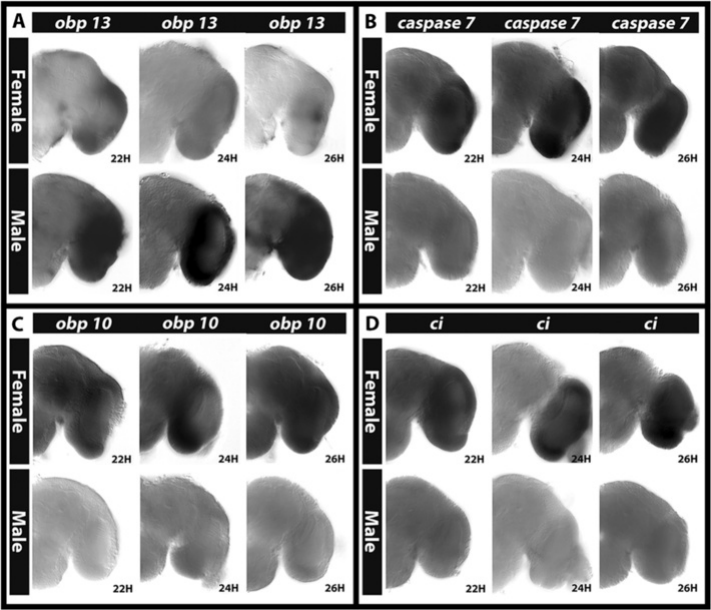
**Sex-specific gene expression patterns are maintained from 22 to 26 h of pupal brain development.** Differential male vs. female *obp13***(A)**, *caspase7***(B)**, *obp10***(C)**, and *ci***(D)** expression patterns in the brain identified at 24 h APF were also confirmed at 22 and 26 h APF. Brain hemisegments are oriented dorsal upward in each panel.

Whole mount *in situ* hybridization was performed to assess the expression of a subset of DETs in male vs. female pupal brains. A variety of genes were selected for these studies. Choices included genes linked to significant GO terms and pathways noted above (see further explanation in the Discussion) and genes of interest to our laboratory. Several genes were selected on their basis of their proximity to Dsx binding sites (see below). First, we performed whole mount *in situ* hybridization experiments in an effort to validate the data set. Differential gene expression was confirmed in the pupal brain for *takeout*, *ci*, *cdk4/6*, *obp10*, *arrow*, *caspase 7* (Figure 4A,B,C,D,E,F; all upregulated in females), *synj*, *geko*, *p53*, and *obp13* (Figure 4G,H,I,J; all upregulated in males). For all of these genes, the female or male sex-specific upregulation detected through *in situ* hybridization in the 24 h pupal brain (Figure 4) was in agreement with the sex-biased expression results detected in the whole head microarray experiments (Additional file 1). To be certain that the dimorphic expression observed did not result from slight age differences between female and male pupal samples, sexually dimorphic patterns of gene expression were also confirmed at both 22 and 26 h of development for all of these genes; representative data for *obp13* (Figure 5A), *caspase 7* (Figure 5B), *obp10* (Figure 5C), and *ci* (Figure 5D) are shown.

**Figure 6:**
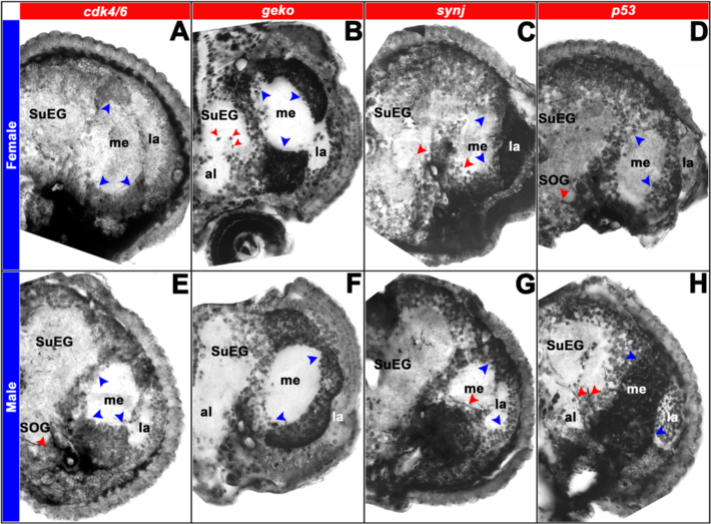
**Sex-specific expression patterns of DETs in sectioned*A. aegypti*pupal brains.** Differential expression of *cdk4/6***(A, E)***, geko***(B, F)***, synj***(C, G)**, and *p53***(D, H)** was detected in 12 μ sections through 24 hr female **(A-D)** and male **(E-H)** pupal brains. Hemisegments oriented dorsal upward are shown. Hybridizations with a sense control probe detected no signal in comparable brain sections (not shown). *cdk4/6* is commonly expressed in the optic lobe (blue arrowheads in **A**, **E**), but males have an additional *cdk4/6* expression domain in the ventral suboesophageal ganglion (red arrowhead in **E**). *geko*, which is commonly expressed in the female and male optic lobe (blue arrowheads in **B**, **F**), is expressed in additional large cell bodies near the female midbrain and antennal lobe (red arrowheads in **B**). *synj* expression is detected in the optic lobe (blue arrowheads in **C**, **G**) and in a subset of midbrain neurons (red arrowheads in **C**, **G**). The red arrowhead in **G** marks sex-specific *synj* optic lobe expression in males, and midbrain *synj* levels are generally higher in males (compare expression adjacent to red arrowheads in **C**, **G**). *p53* is expressed in the optic lobe and suboesophageal ganglion of females (blue and red arrowheads, respectively in **D**). *p53* is also expressed in the male optic lobe (blue arrowheads in **H**), but not in the male subesophageal ganglion **(H)**. Male-specific *p53* expression is detected in neurons adjacent to the antennal lobe (red arrowheads in **H**). These data are consistent with the results presented in Figure 7. *al* antennal lobe, *la* lamina, *me* medulla, *SuEG*, supraesophageal ganglion.

For many of the genes analyzed, dimorphic expression localized to the optic lobe of the brain. For example, *takeout*, *ci*, *obp10*, *arrow*, and *caspase 7* (Figure 4A,B,D,E,F, respectively) are upregulated in the female optic lobes with respect to males in which little if any transcripts were detected through *in situ* hybridization. Expression of *obp13* is upregulated in the male optic lobe with respect to females (Figure 4J). To be certain that this intense optic lobe staining was not an artifact of the tissue preparation or whole mount *in situ* hybridization process, we confirmed that negative sense control probes gave little background staining in the optic lobes or in other regions of the brain (Figure 4K). We also confirmed optic lobe expression for a number of DETs by performing *in situ* hybridization experiments on sectioned brain tissues (Figure 6).

**Figure 7:**
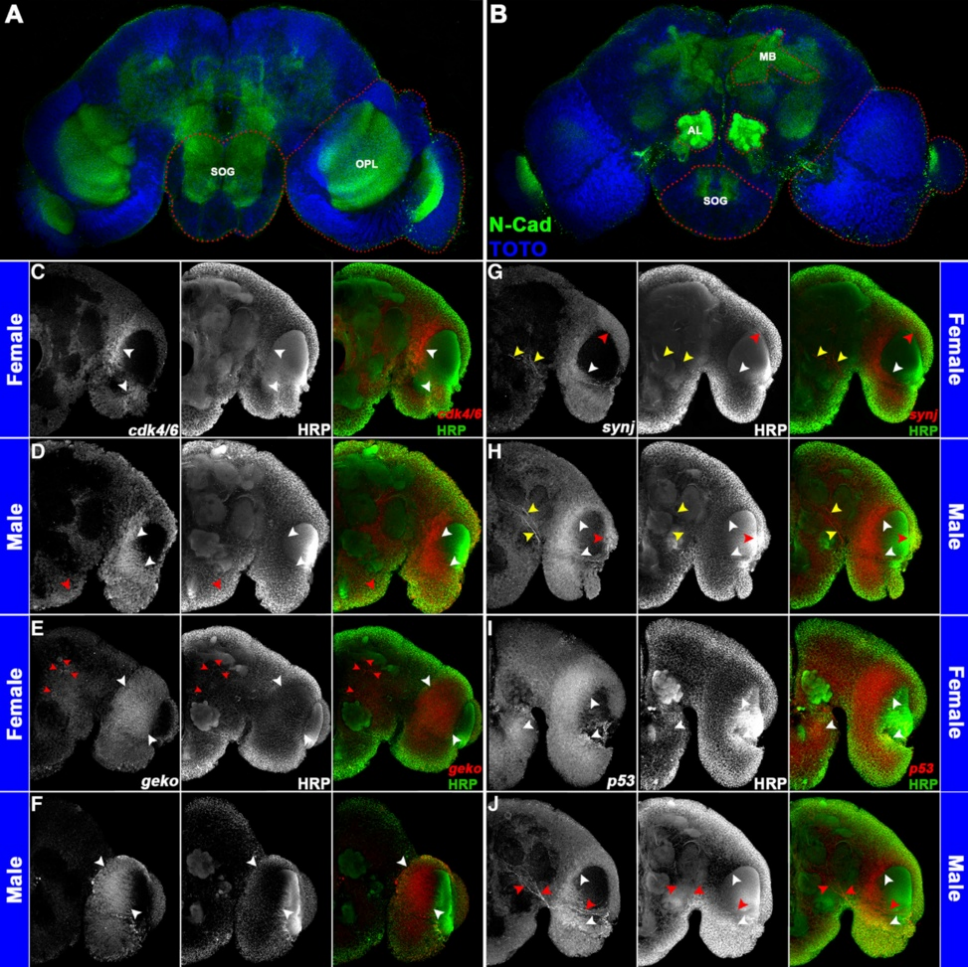
**Sex-specific expression patterns of DETs in the*A. aegypti*pupal brain.** The optic lobe (OPL), antennal lobe (AL), suboesophageal ganglion (SOG), and mushroom body (MB) regions are highlighted by red dots in a whole brain labeled with anti-N-Cadherin (green in **A**, **B**) and TOTO nuclear stain (blue in **A**, **B**). These regions were assessed through confocal imaging following whole mount *in situ* hybridization and anti-HRP staining. Five merged Z-stacks (totaling 5 μ) of brain hemisegments oriented dorsal upward **(C-J)** labeled with anti-HRP staining (center panels in **C**-**J**; green in overlays at right) and riboprobes corresponding to the indicated transcripts (left panels in **C**-**J**; red in overlays at right) are shown. Differential expression of *cdk4/6***(C, D)**, *geko***(E, F)**, *synj***(G, H)**, and *p53***(I, J)** was detected in 24 h female **(C, E, G, I)** and male **(D, F, H, J)** pupal brains. *cdk4/6* is commonly expressed in the optic lobe (white arrowheads in **C**, **D**), but males have an additional *cdk4/6* expression domain in the ventral suboesophageal ganglion (red arrowhead in **D**). *geko*, which is commonly expressed in the female and male optic lobe (white arrowheads in **E**, **F**), is expressed in additional large cell bodies near the female midbrain and antennal lobe (red arrowheads in **E**). *synj* expression is detected in the optic lobe (white/red arrowheads in **G**, **H**) and in a subset of midbrain neurons (yellow arrowheads in **G**, **H**). Red arrowheads mark sex-specific *synj* optic lobe expression **(G, H)**, and midbrain *synj* levels are generally higher in males (compare expression adjacent to yellow arrowheads in **G**, **H**). *p53* is expressed in the optic lobe and suboesophageal ganglion of females (white arrowheads in **I**). *p53* is also expressed in the male optic lobe (white arrowheads in **J**), but not in the male subesophageal ganglion. Male-specific *p53* expression is detected in neurons adjacent to the antennal lobe (red arrowheads in **J**). These data are consistent with the results presented in Figure 6.

Experiments on sectioned brain tissue (Figure 6), as well as co-labeling experiments with anti-HRP, which marks various neuropil regions in the developing mosquito brain [23] (Figure 7C,D,E,F,G,H,I,J), helped us to pinpoint the location of dimorphic gene expression for a number of DETs in which dimorphic expression was noted outside of the optic lobe region. These studies revealed upregulation of *cdk4/6* expression in the ventral region of the male subesophageal ganglion (Figure 6E, compare to Figure 6A; Figure 7D, compare to Figure 7C), as well as upregulation of *geko* transcripts near the midbrain and antennal lobes of females (Figure 6B, compare to Figure 6F; Figure 7E, compare to Figure 7F). Higher levels of *synj* expression were detected in the midbrain of males (Figure 6G, compare to Figure 6C; Figure 7H, compare to Figure 7G), and sex-specific patterns of *synj* expression were detected in the optic lobe (Figures 6C,G and 7G,H). Finally, *p53* is expressed in the female, but not the male subesophageal ganglion (Figure 6D, compare to Figure 6H; Figure 7I, compare to Figure 7J), while male-specific *p53* expression was detected in neurons adjacent to the antennal lobe region (Figures 6H and 7J).

### 3.3 Putative Dsx consensus binding sites associated with DETs

The consensus binding site sequence for Dsx, GCAACAATGTTGC, was deduced by Luo et al. [28], who reported that the sequence of this binding site is well-conserved in many dipteran species and is detected 26-fold over expected in *A. aegypti.* Both male and female Dsx splice variants share this DNA-binding domain but have different C termini that differentially direct sex-specific gene expression [11],[12],[16]. Search of the *A. aegypti* genome for the Dsx consensus sequence uncovered 732 genes located in proximity to putative Dsx binding sites (Additional file 2). *A. aegypti* genes with intragenic, upstream, or downstream Dsx consensus sites grouped under a number of significant GO-terms, which are itemized in Table 5. Many of these are linked to neurological processes or neural development. These include a variety of GO terms related to the sensory system and sensory development, particularly the compound eye and compound eye development. Additional neural GO terms associated with these genes include neuron development, neuron differentiation, neuron fate commitment, and neurological system processes. Moreover, 48 of the 732 genes associated with Dsx binding sites correspond to genes identified in the dimorphism microarray experiments (Additional file 2). Together, these data support the hypothesis that Dsx is a regulator of sexually dimorphic gene expression in the *A. aegypti* nervous system. This hypothesis was examined in the developing *A. aegypti* pupal brain.

**Table 5:** Over-represented GO categories among genes associated with Dsx consensus binding sites

**Term**	**Count**	***p*** **value**
GO:0007166—cell surface receptor linked signal transduction	14	0.026157815
GO:0030182—neuron differentiation	12	0.00682396
GO:0007423—sensory organ development	12	0.006946573
GO:0003002—regionalization	11	0.034377574
GO:0007389—pattern specification process	11	0.04735023
GO:0001745—compound eye morphogenesis	10	0.002178058
GO:0048592—eye morphogenesis	10	0.003563147
GO:0048749—compound eye development	10	0.008521908
GO:0001654—eye development	10	0.013504773
GO:0048666—neuron development	10	0.01756628
GO:0007350—blastoderm segmentation	8	0.01232515
GO:0009880—embryonic pattern specification	8	0.016503315
GO:0035282—segmentation	8	0.029278118
GO:0001751—compound eye photoreceptor cell differentiation	7	0.001660725
GO:0001754—eye photoreceptor cell differentiation	7	0.00207928
GO:0046530—photoreceptor cell differentiation	7	0.003406961
GO:0048232—male gamete generation	7	0.008583448
GO:0007283—spermatogenesis	7	0.008583448
GO:0045165—cell fate commitment	7	0.049850932
GO:0022604—regulation of cell morphogenesis	6	0.011632368
GO:0042051—compound eye photoreceptor development	5	0.003084585
GO:0042462—eye photoreceptor cell development	5	0.003302533
GO:0042461—photoreceptor cell development	5	0.005753226
GO:0009968—negative regulation of signal transduction	5	0.029343023
GO:0010648—negative regulation of cell communication	5	0.03026886
GO:0040007—growth	5	0.037232894
GO:0048193—Golgi vesicle transport	4	0.012925753
GO:0016049—cell growth	4	0.017389354
GO:0046552—photoreceptor cell fate commitment	4	0.020430767
GO:0048663—neuron fate commitment	4	0.02374654
GO:0007507—heart development	4	0.044413566
GO:0007479—leg disc proximal/distal pattern formation	3	0.01405488
GO:0035223—leg disc pattern formation	3	0.01405488
GO:0035051—cardiac cell differentiation	3	0.021604074
GO:0035287—head segmentation	3	0.023702413
GO:0007449—proximal/distal pattern formation, imaginal disc	3	0.023702413
GO:0009954—proximal/distal pattern formation	3	0.028137457
GO:0035215—genital disc development	3	0.040521397
GO:0006869—lipid transport	3	0.040521397
GO:0045466—R7 cell differentiation	3	0.045952969
GO:0007419—ventral cord development	3	0.048763797
GO:0048052—R1/R6 cell differentiation	2	0.034007252
GO:0007462—R1/R6 cell fate commitment	2	0.034007252
GO:0031887—lipid particle transport along microtubule	2	0.045086826

*A. aegypti* genes associated with Dsx consensus sites (defined as genes with Dsx consensus sites located within or 10 kB upstream/dowstream of the genes) grouped under a number of significant GO terms (*p* < 0.05). The number of genes associated with each GO term (count) and corresponding Fisher's exact test *p* values are indicated.

### 3.4 Dsx is a regulator of sex-specific gene expression in the developing *A. aegypti* pupal brain

**Figure 8:**
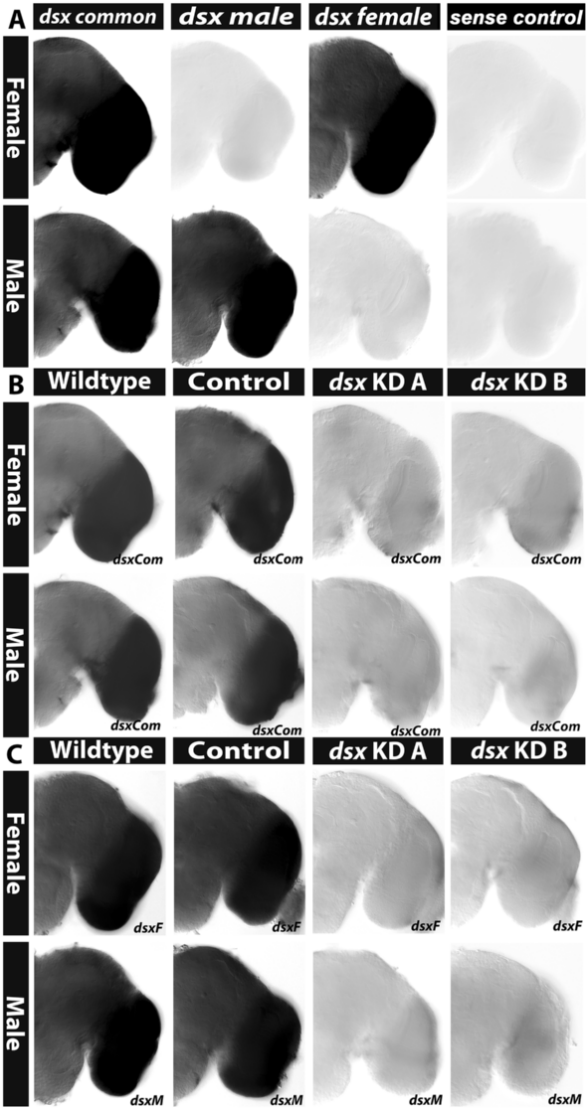
**Expression and knockdown of*dsx*in the*A. aegypti*pupal brain. (A)** Detection of *dsx* expression in developing wild-type female (top) and male (bottom) pupal brain hemisegments with riboprobes corresponding to exon 2 (*dsx* common), DsxM *(dsx* male), and DsxF *(dsx* female). At right in **(A)**, a brain processed with a sense control probe lacks staining. Knockdown of both splice variants was detected through use of the *dsx* common **(B)**, *dsxF* (**C**, top), and *dsxM* (**C**, bottom) probes in female (top in **B**, **C**) and male (bottom in **B**, **C**) pupal brain hemisegments following injection of *dsx KD A* or *KD B* siRNAs. Control siRNA-injected animals (control in **B**, **C**) resemble wild-type animals. Brain hemisegments are oriented dorsal upward in each panel.

**Figure 9:**
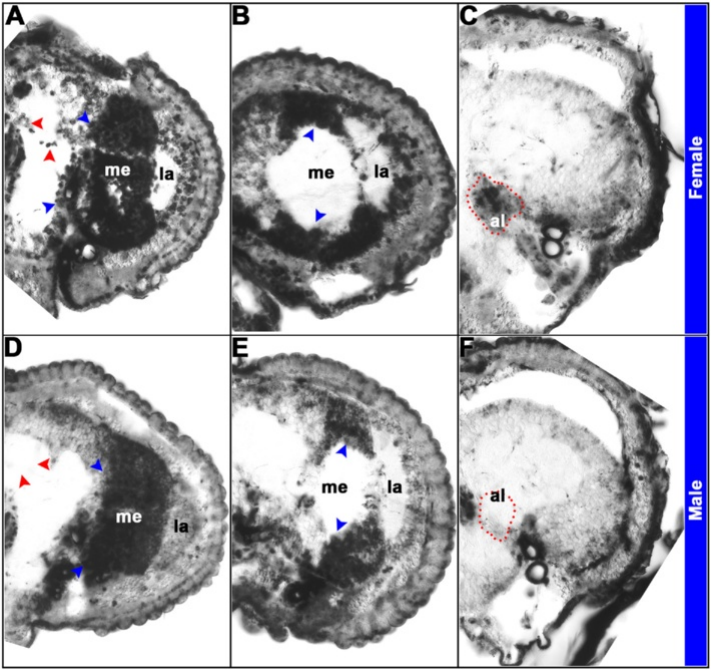
**Sex-specific expression patterns of*dsx*in sectioned*A. aegypti*pupal brains.** The detailed expression pattern of *dsx* was analyzed through *in situ* hybridization experiments (using the *dsx* common probe) that were performed following preparation of paraffin sections of female **(A-C)** and male **(D-F)** heads. Twelve micron sections through different portions of the brain reveal the following structures, which are shown in brain hemisegments oriented dorsal upward in **(A-F)**: antennal lobe (al), lamina (la), and medulla (me). Expression of *dsx* is detected in the visual system of both females and males (blue arrowheads in **A**, **B**, **D**, **E**). However, *dsx* is dimorphically expressed in the antennal lobe (highlighted by red dots in **C**, **F**) and the mushroom bodies (red arrowheads in **A**, **D**) of females and males. Hybridizations with a sense control probe detected no signal in comparable brain sections (not shown). These data are consistent with the results presented in Figure 10.

**Figure 10:**
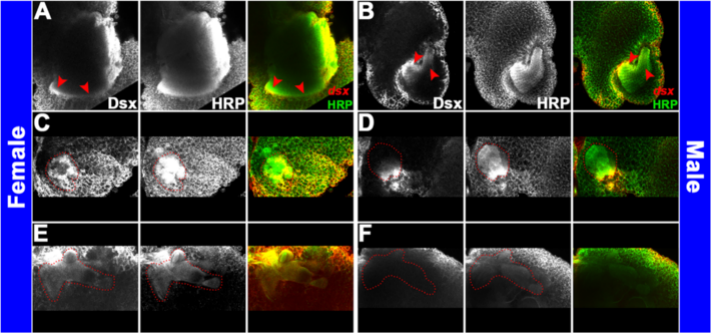
**Sex-specific expression patterns of*dsx*in the*A. aegypti*pupal brain.** The precise locations of *dsx* expression in the 24 h female **(A, C, E)** and male **(B, D, F)** pupal brain were also mapped through co-labeling experiments with *dsx* common probe (left in panels **A**-**F**; red in overlays at right) and anti-HRP staining (center panels of **A**-**F**; green in overlays at right), which marks various neuropil regions in the developing *A. aegypti* brain. Confocal imaging was performed following staining of whole mount brains. Five merged Z-stacks of confocal sections (totaling 5 μ) through the optic lobe **(A, B)**, antennal lobe **(C,D)**, and mushroom body **(E, F)** regions of brain hemisegments oriented dorsal upward are shown. *dsx* expression is detected in the 24 h female pupal optic lobe **(A)**, throughout the antennal lobe **(C)**, and in the mushroom body **(E)**. *dsx* expression is detected in the 24 h male pupal optic lobe **(B)** and a group of ventral-most cells in the antennal lobe **(D)**, but not in the mushroom body **(F)**. These data are consistent with the results presented in Figure 9.

Sex-specific *dsx* expression was examined in the *A. aegypti* pupal brain. For these experiments, probes corresponding to the male and female splice forms, as well as exon 2 which is common to both splice forms [16] (see Methods for details) were synthesized and utilized in whole mount *in situ* hybridization experiments. Expression of the male splice form was detected in the male brain, and expression of the female-specific splice form was detected in the female brain, while the *dsx* common probe marked expression of *dsx* transcripts in both female and male pupal brains (Figure 8A). In females and males, the highest levels of *dsxF* and *dsxM* transcripts, respectively, are detected in the optic lobes (Figure 8A). To more precisely map the locations of sex-specific *dsx* expression outside of the optic lobe region, we again performed *in situ* hybridization experiments on sectioned head tissue (Figure 9) and also performed co-labeling experiments with anti-HRP antibody (Figure 10). These experiments revealed dimorphic expression patterns of *dsx* in the *A. aegypti* antennal lobe and mushroom body. Expression of *dsx* is detected in the 24 h female antennal lobe (Figures 9C and 10C). In males, although *dsx* expression is detected in the antennal lobe (Figures 9F and 10B), it is restricted to the ventral-most cells. Furthermore, although *dsx* expression is detected in the female mushroom body (Figures 9A and 10E), *dsx* expression is not detected in the male mushroom body (Figures 9D and 10F). These expression studies suggest that *dsx* may regulate sex-specific gene expression in the *A. aegypti* pupal brain. We conducted *dsx* knockdown experiments to test this hypothesis.

siRNA-mediated gene targeting was used to knockdown *dsx* in *A. aegypti* pupae (Figure 7B,C). Two *dsx* knockdown siRNAs, *dsx KD A* and *dsx KD B*, which correspond to two different target sequences in exon 2, an exon common to both the female and male splice forms, were used in these experiments. *dsx KD A* and *dsx KD B* effectively target pupal brain expression of both the male and female *dsx* splice forms when microinjected into the pupal thorax (Figure 8B,C). In these experiments, control injections were performed with a scrambled version of *dsx KD B*, an siRNA which lacks significant sequence homology to any genes in the *A. aegypti* genome (Figure 8B,C).

**Figure 11:**
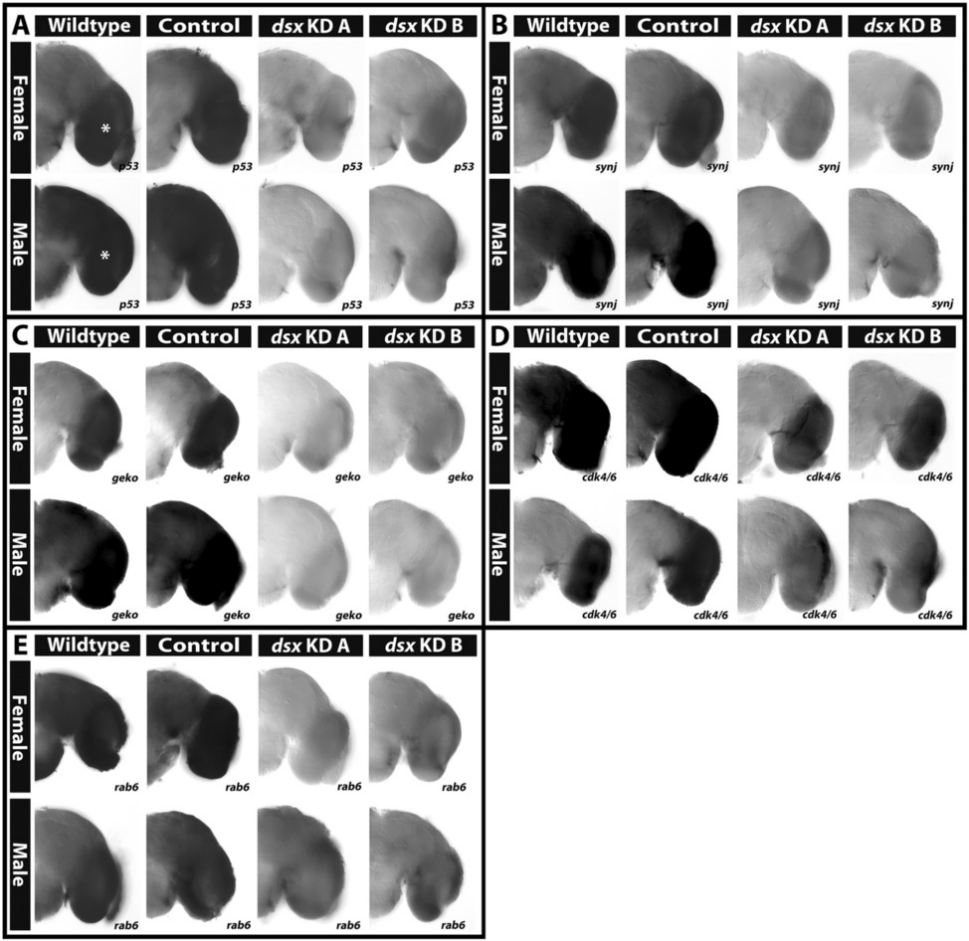
**Dsx is a regulator of sex-specific gene expression in the*A. aegypti*pupal brain.** Sex specific patterns of *p53***(A)**, *synj***(B)**, *geko***(C)**, *cdk4/6***(D)**, and *rab6***(E)** mRNA transcripts were detected in female (upper portion of each panel) and male (lower portion of each panel) wild-type and control-fed 24 h pupal brain hemisegments, which are oriented dorsal upward in each panel. The position of the optic lobe is marked by an asterisk in **(A)** and located at a similar position in the other panels. The sex-specific expression patterns of each gene were disrupted following injection of either *dsx KD A* or *KD B* siRNAs.

The impact of *dsx* knockdown on the developmental expression of putative Dsx target genes was then assessed. *p53*, a DET in the microarray study that is upregulated in male heads (Additional file 1), is associated with a Dsx binding site (Additional file 2) and is differentially expressed in wild-type and control-fed female and male pupal brain hemisegments (Figures 4I and 11A). Whole mount *in situ* hybridization experiments revealed that levels of *p53* are reduced to nearly undetectable levels in female and male pupae injected with *dsx KD A* or *dsx KD B* siRNAs (Figure 11A). Comparable results were obtained for *synj* (Figures 4G and 11B), *geko* (Figures 4H and 11C), and *rab6* (Figure 11E), all of which are genes associated with Dsx consensus binding sites (Additional file 2) that correspond to DETs noted in microarray studies (Additional file 1). Expression of *cdk4/6* (Figures 4C and 11D), which contains a Dsx binding site, was reduced but not completely eliminated in *dsx* knockdown females and males, in which *cdk4* transcripts could still be detected in the optic lobes, albeit at reduced levels. Together, these results indicate that Dsx is required for sex-specific gene expression in the developing *A. aegypti* pupal central nervous system.

## 4 Discussion

### 4.1 Sex-specific gene expression in the developing *A. aegypti* pupal head

This investigation revealed global patterns of sex-specific gene expression in the *A. aegypti* pupal head transcriptome, which was assessed through custom *A. aegypti* microarray experiments (Additional file 1). These studies provided insight regarding the genes, pathways, and processes impacted by sex-specification in the 24 h pupal head transcriptome. The data collected may be of interest to those studying a number of tissues of vector importance, including the fat bodies, antennae, maxillary palp, and proboscis. However, the primary focus of the present study is the brain, and so the results of the microarray experiments were validated through *in situ* hybridization experiments performed on female and male pupal brain tissue (Figures 4, 5, 6, 7, 8, 9, 10). The 24 h pupal brain was selected, as our recent work has identified this time point to be a critical period for nervous system development. At this stage, which follows periods of extensive proliferative activity and pupal histolysis, the brain has an anatomical organization structure that is similar to that of the adult *A. aegypti* brain. The neuropils that are characteristic of the adult brain, including the antennal lobe, central complex, and three optic lobe neuropils, have begun to form. At 24 h APF, extensive neural process outgrowth, targeting of higher order brain neurons, synapse formation, and arborization occur, ultimately generating the increased neuropil density observed in the adult [6],[8],[23].

These studies detected sexually dimorphic expression of *obp 10* and *takeout* through both head transcriptome microarray experiments and through analysis of transcript expression in the developing brain (Additional file 1, Figure 4A,D). Although expression of these genes had not previously been spatially assessed in the developing brain, dimorphic expression of both genes had been noted in *A. aegypti* by Bohbot and Vogt [31], who hypothesized that the genes might regulate differential olfactory and feeding behaviors in mosquitoes. It was subsequently demonstrated through knockdown experiments in *Anopheles gambiae* that Takeout regulates blood-feeding propensity [32]. Our own ability to detect dimorphic expression of these genes further validates the effectiveness of the methodological approach employed, while also raising the question of whether their dimorphic expression in the brain is functionally significant. This is a topic to be explored in future studies, which would be greatly enhanced through technical advancements that permit the manipulation of gene expression in a brain-specific manner in mosquitoes.

Components of critical developmental signaling cascades, including *cubitus interruptus (ci)*, a member of the Hedgehog signaling pathway (reviewed by [33]), as well as *arrow*, a component of the Wnt/Wingless (Wg) signaling pathway [34], are upregulated in the developing female pupal brain (Figure 4B,E, Additional file 1). It will be interesting to functionally examine the roles of these genes during brain development, particularly given that *Drosophila* Ci has both transcriptional activation and transcriptional repressor forms (reviewed by [33]) that might differentially shape male and female development. Furthermore, although the function of Wg signaling in the regulation of sex-specific development has not yet been assessed in the insect nervous system, Wg signaling has been shown to regulate sexually dimorphic abdomen development in *Drosophila.* In flies, dimorphic Wg regulation in conjunction with monomorphic segment-specific cell death generates sex-specific abdomen shape [35].

More broadly, the results of this investigation suggest that underlying differences in proteolytic, metabolic, catabolic, and biosynthetic processes, as well as cell growth/proliferation and ion transport underlie differences in *A. aegypti* males vs. females (Tables 1, 2, 3, 4; Figures 2 and 3). Here, we discuss the potential relevance of these findings, focusing this discussion on development of the nervous system.

### 4.2 Proteases

Proteases, especially serine proteases, are significantly upregulated in female pupal heads (Figure 3, Table 2). These include 23 Clip-domain serine proteases that are significantly overexpressed in females. Clip-domain serine proteases have been well studied with regard to their multiple roles in insect immunity, including antimicrobial peptide synthesis, hemolymph coagulation, and melanization of pathogen surfaces [36]. In *Anopheles gambiae*, Clip-domain proteases are known for their function in the regulation of malaria parasite melanization [37]. In reference to insect development, two Clip-domain proteases function as components of the Toll pathway in the regulation of dorso-ventral axis specification (reviewed in [38]). Although Clip proteases have not been well studied in the context of the developing nervous system, the results of this investigation suggest that they are dimorphically expressed in female and male pupal heads. Other types of serine proteases have important CNS functions, including the regulation of cell migration, neurite outgrowth, and pathfinding, glial and neuronal cell survival, and synaptic remodeling [39]-[42]. Thus, it is possible that these Clip proteases might function as modifiers of the extracellular matrix in the context of neural migration.

### 4.3 Cell cycle/cell death

Cell cycle components (Figure 2A) were noted as a significant GO term among DETs upregulated in females (Table 1). For example, *cdk4/6*, a known regulator of cellular growth in *D. melanogaster*[43],[44], is upregulated in female pupal heads (Figures 4C and 11D). In flies, overexpression of Cyclin D-Cdk4/6 promotes cellular growth, and loss of *cdk4* function results in flies with a smaller body size [43],[44]. It is possible that increased expression of *cdk4* in *A. aegypti* females upregulates the growth of *A. aegypti* female pupae/heads with respect to males, which have a significantly smaller body and head (but not brain) size [18]. In relation to the developing nervous system, it has been shown that *cdk4* expression is positively regulated in response to axon guidance gene signaling [45]. Thus, upregulation of *cdk4* in the developing *A. aegypti* female brain might contribute to differential neural outgrowth. Furthermore, the microtubule-based movement GO term was significantly associated with DETs upregulated in females (Figure 2A; Table 1), suggesting that spindle fiber movement may be regulated in a sex-specific manner.

*p53*, a regulator of G1-S transition (reviewed by [46]), is also differentially expressed in pupal heads (Additional file 1), in which increased *p53* expression can be detected in the male 24 h pupal brain (Figures 4I and 11A). In flies, p53 regulates apoptosis during development (reviewed by [46]). The regulation of cell death is a critical aspect of nervous system development which has also been linked to the development of sexual dimorphism in fruit flies (reviewed by [9]). For example, Dsx regulates death of the female TN1 neurons during *Drosophila* metamorphosis. In males, these neurons survive and are believed to function in the neural circuitry that is involved in production of the male courtship song (reviewed by [9]). These findings, in conjunction with the results of our investigation, suggest that regulation of *p53* expression by Dsx may generate differences in programmed cell death that might shape the development of sex-specific traits in mosquitoes. In further support of this notion, *caspase 7*, an ortholog of *Drosophila death caspase 1* and *Ice*, was also found to be expressed in a sex-specific manner (Figure 4F; Additional file 1). In addition to regulation of apoptosis, p53 has also been shown to regulate neurite outgrowth and axonal regeneration [47], suggesting that it may impact these processes in the mosquito brain. Finally, p53 has been linked to the regulation of life span in *Drosophila*[48]. Overexpression of p53 in the female nervous system results in increased life span in females. Thus, it is possible that female-specific upregulation of *p53* expression in the *A. aegypti* female brain may contribute to the relatively longer lifespan of *A. aegypti* females with respect to males.

### 4.4 Ion transport

A wide variety of ion transporters are upregulated in male pupal heads (Figure 2B, Table 4), which suggests that sexually dimorphic differences in the developing mosquito brain may result from differential ion channel expression. As discussed by Yamamoto and Lopez-Bendito [49], the interaction of neural activity and genetic programs specifies neural circuit composition and organization during development. Changes in electrical activity regulate neural growth, axonogenesis, and the branching of axons. The sex-specific differences in ion transport-related genes noted here are therefore of potential interest with respect to the development of sexually dimorphic neural circuitries in mosquitoes. Furthermore, the activation and inactivation properties of ion channels in axons determine the short-term dynamics of axonal propagation and synaptic transmission [50].

Our microarray experiments detected upregulated male-specific expression of *glutamate receptor 7* (Table 4), an ortholog of *D. melanogaster ionotropic receptor (IR) 25a.* In relation to this, glutamate metabolism was also noted as a significant pathway among DETs upregulated in males (Figure 3; Additional file 3). In *Drosophila*, IRs, which have evolved from ionotropic glutamate receptors (iGluRs), are expressed in a combinatorial fashion in sensory neurons and respond to many distinct odors [51]. Sensory neurons expressing *IR84a* in fruit flies innervate a glomerulus that expresses male-specific isoforms of the sex determination gene *fruitless (fru)*, one of three sexually dimorphic glomeruli in the antennal lobe. Mutation of *IR84a* reduces male courtship (reviewed by [52],[53]). These observations suggest that differential expression of glutamate receptors and differential glutamate metabolism may impact sex-specific behaviors in mosquitoes.

### 4.5 Proteasome

DETs upregulated in females were significantly associated with the proteasome (Figure 3, Additional file 3), which is well known for its role in the intracellular degradation of ubiquitinated proteins. Protein synthesis and degradation are particularly important to neuronal development and function because of the large distances between synapses and soma. The ubiquitin proteasome system influences synaptic protein levels through the regulation of protein function and localization, as well as endocytosis (reviewed by [54]). It should be noted that *synj* (Figures 4G, 6C,G, 7G,H, 11B, Additional file 1), which also regulates endocytosis at the *Drosophila* synapse [55], is dimorphically expressed in the *A. aegypti* pupal brain. Elements of the ubiquitin proteasome system have also been found to regulate synaptic strength, homeostatic plasticity, axon growth and guidance, and dendrite morphogenesis (reviewed by [54]). Thus, differential expression of ubiquitin proteasome pathway components in female and male mosquitoes may have significant impacts on neural development and function that contribute to the development of sexually dimorphic behaviors.

### 4.6 Biosynthetic and catabolic processes

Significantly upregulated DETs in the female pupal head were also associated with biosynthetic and catabolic processes (Figure 3, Additional file 3). For example, glycosphingolipid biosynthesis is significantly over-represented among DETs upregulated in females. The functions of glycosphingolipids have been well studied in the context of development and neurobiology (reviewed by [56],[57]). For example, gangliosides, sialic acid-containing glycosphingolipids, are abundant in the nervous system, where they function in cell-cell recognition, adhesion, and signal transduction [57]. Work in *Drosophila* has shown that sphingolipid regulators affect cell survival, growth, and specification, as well as the control of lipid storage and responses to nutrient availability [56]. Thus, the differential regulation of sphingolipids in the developing male and female brain could have significant impact on the development of sex-specific behaviors in *A. aegypti.*

DETs significantly upregulated in female pupal heads are also associated with the glycosaminoglycan (GAG) degradation pathway (Figure 3, Additional file 3). In *A. aegypti*, cell surface GAGs have primarily been studied in relation to their potential roles as dengue virus receptors (reviewed by [58]). It will therefore be interesting to determine if there is differential GAG expression in other tissues, such as the midgut, a subject for future investigations. In reference to the brain, extracellular proteoglycans, which have one or more covalently attached GAGs, are known to impact many aspects of neural development and CNS maintenance. For example, during development, proteoglycans regulate cell adhesion, neurite formation, axon growth and guidance, and synapse formation. Proteoglycans have also been implicated in axon regeneration and sprouting following injury [59],[60]. Thus, differential GAG degradation could impact multiple aspects of neural development and function in *A. aegypti* females vs. males.

### 4.7 Dsx is a regulator of dimorphic gene expression in the *A. aegypti* pupal nervous system

Our search of the *A. aegypti* genome for the Luo et al. [28] Dsx consensus sequence uncovered 732 putative Dsx binding sites (Additional file 2), 48 of which correspond to genes identified in our microarray experiments (Additional file 3). Our search algorithm allowed for one mismatch in the consensus binding site (see Methods). This was useful to us since it permitted the most rigorous selection of putative targets prior to pursuing the *dsx* knockdown studies, which are labor-intensive. However, it is quite possible/probable that more mismatches are permissible and that additional binding sites are present in the genome. To more fully pursue the identification of Dsx binding sites throughout the genome, future studies might include a ‘wet lab’ approach, for example chromatin immunoprecipitation with anti-Dsx antibodies, or perhaps through use of the DamID approach utilized by Luo et al. (2011) to assess *D. melanogaster* Dsx binding sites.

Few of the targets identified in our study correspond to known Dsx transcriptional targets in *D. melanogaster*[28], suggesting that the targets of Dsx have evolved rapidly within the Diptera and highlighting the need to pursue analysis of *dsx* function in mosquitoes. To this end, we used siRNA-mediated gene targeting to examine the function of *dsx* in the developing pupal brain (Figure 11). These experiments indicated that Dsx is a key regulator of sex-specific gene expression during *A. aegypti* neural development. The detection of Dsx consensus binding sites associated with the *p53*, *synj*, *geko*, *cdk4/6*, and *rab6* genes (Additional file 2), all of which have sexually dimorphic expression in the pupal brain (Additional file 1, Figures 4, 6, 7, 11) that is disrupted by *dsx* knockdown (Figure 11A-E), suggests that Dsx directly regulates expression of these genes. However, Dsx of course may function to regulate expression of these genes and its other targets through direct and/or indirect mechanisms, a question for future studies. Moreover, studies of the regulation of yolk protein genes, which are direct targets of Dsx in *D. melanogaster*, suggested that DsxF acts as a transcriptional activator, while DsxM functions as a repressor [61],[62]. Interestingly, none of our *dsx* knockdown experiments in *A. aegypti* resulted in upregulation of putative target gene expression in males or females (Figure 11), but it will be interesting to explore this subject further in future investigations.

The roles of *dsx* in development of the nervous system have been explored in *D. melanogaster.* In flies, Dsx regulates differences in programmed cell death, which is known to contribute to sexually dimorphic nervous system development [9]. It is therefore possible that control of *p53* expression by Dsx in the *A. aegypti* CNS (Figure 11A) may be required for regulation of sex-specific cell death patterns in the mosquito brain, an interesting subject for future investigations. Dsx has also been associated with dimorphic cell proliferation in the *Drosophila* CNS, which is interesting in light of the observation that Dsx regulates expression of the cell cycle regulator *cdk4/6* in the developing *A. aegypti* brain (Figure 11D). Moreover, Rideout et al. [63] detected sex-specific differences in the numbers, axonal projections, and synaptic density of Dsx-expressing neurons in *Drosophila*, and Dsx may regulate these aspects of neural development in mosquitoes. Finally, a recent study demonstrated that in female fruit flies, the neural circuitry associated with female post-mating behavior is specified by Dsx function. During copulation, this female circuitry senses male sex peptides and relays the signal to higher order circuits in the brain that generate post-mating physiological and behavioral output responses in females [64]. It will be interesting to determine if Dsx plays similar roles in mosquitoes.

We note that in comparison to *D. melanogaster*, in which sex-specific expression of *dsx* is detected in only several subsets of neurons [63],[65], *dsx* is expressed much more broadly within the *A. aegypti* female and male pupal brain (Figures 8A, 9, 10). For example, although *dsx* expression is not detected in the optic lobe of the *D. melanogaster* pupal brain, *A. aegypti dsx* is expressed in the male and female pupal optic lobes (Figures 8A, 9A,D, 10A,B). Moreover, we detect sex-specific differences in *dsx* expression in the antennal lobe (Figures 9C,F, 10C,D) and mushroom bodies (Figure 9A,D, 10E,F) of *A. aegypti*, differences which have not been reported in *D. melanogaster* pupae. These findings suggest that Dsx may play a prominent role in the regulation of sex-specific neural development in *A. aegypti,* in which its dimorphic expression pattern suggests that it may contribute to sex-specific differences in the visual and olfactory systems, the processing of sensory information, as well as learning and memory.

Previous studies have demonstrated that Dsx and Fru act in the same neurons to generate neuronal wiring and behaviors [63],[66],[67]. Fru targets, which were recently identified in *D. melanogaster*[68],[69], include many genes that regulate neural processes, including neurotransmission, ion-channel signaling, and neuron development. Furthermore, Neville et al. [68], who detected over-representation of known Dsx targets in their FruM target data set, speculate that *Drosophila* Dsx and Fru act together, either in a physical complex or through coregulation of target genes, to specify sex-specific neural development. Sex-specific Fru splice forms have been detected in *A. aegypti*[70], but the expression patterns of these transcripts have not yet been reported in the developing brain, and *fru* function has not been investigated in mosquitoes. In future studies, it will be interesting to functionally assess the roles of *fru* isoforms during *A. aegypti* female and male neural development.

### 4.8 Sexual dimorphism in the visual system

Differential expression of a number of the transcripts detected in the sex-specific pupal head transcriptome localized to the optic lobe of the brain (Figures 4 and 11), where high levels of the female and male *dsx* splice forms were also detected (Figures 8A, 9A,D, 10A,B). Moreover, many of the over-represented GO categories among genes with Dsx consensus binding sites are related to the compound eye or compound eye development (Table 5). Combined, these findings suggest that male and female compound eyes and higher order visual processing may differ in *A. aegypti.* Such differences could potentially contribute to female-specific behaviors, such as detection of blood meal hosts or oviposition sites. It is also possible that dependence on vision for mating success differs in males and females. This has been observed in *D. melanogaster*, in which males have a strong dependence on visual function for finding and tracking females, while female receptivity is based on responses to non-visual cues, such as male courtship song quality [71]. Exploration of these topics will prove interesting in future investigations.

Sexually dimorphic visual development has been particularly well-studied in stalk-eyed flies, which as a result of their elongated and sexually dimorphic eye-stalks are a model system for investigating sexual selection [72]. Wilkinson et al. [72] compared sex-specific global patterns of gene expression in *Teleopsis dalmanni*, in which they identified 415 female-biased and 482 male-biased transcripts associated with dimorphic eyestalk development. Many of the genes upregulated in developing female eyestalks are associated with cell differentiation and patterning, which was also the case for genes flanking Dsx consensus binding sites in *A. aegypti* (Table 5). DETs upregulated in males are disproportionately associated with *Teleopsis dalmanni* growth, which is to be expected given the male-specific eye stalk elongation observed in this species. Genes flanking Dsx consensus sites in *A. aegypti* (Additional file 2) are also significantly associated with growth (Table 5), and as discussed above, DETs upregulated in females are associated with the cell cycle (Figure 2). It is likely that these genes are associated with overgrowth of female tissues in *A. aegypti* given that female pupae of this species are larger than males. In support of this notion, *cdk4*, a positive regulator of cellular growth in *D. melanogaster*[43],[44], is upregulated in the *A. aegypti* female pupal brain.

The results of this investigation indicate that *rab6*, which is upregulated in females, is a target of Dsx (Figure 11E). In *Drosophila*, Rab6 functions as a GTP-binding protein regulator of vesicular trafficking that has been implicated in the transport of rhodopsin [73]. It will be interesting to determine if Rab6 expression is dimorphic in the eye and in adult *A. aegypti*, in which it could potentially regulate Rhodopsin transport. The *A. aegypti* genome project uncovered 10 Rhodopsins [1]. Based on studies in *D. melanogaster*, one would expect that changes in Rab6 levels would differentially impact transport of the 10 Rhodopsin proteins [73]. If this is the case in *A. aegypti*, differential Rab6-mediated transport of the 10 Rhodopsin proteins might be of significant consequence to male and female vision. This question, as well as functional analysis of many other DETs in the developing visual system, will be explored in future investigations.

## 5 Conclusions

This investigation revealed sex-specific gene expression profiles in the developing *A. aegypti* pupal head (Figure 1, Additional file 1) and identified Dsx as a key regulator of sexually dimorphic gene expression during mosquito CNS development (Figure 11). Analysis of DETs suggests that dimorphic expression of genes linked to proteolysis, the proteasome, metabolism, catabolic and biosynthetic processes, cell growth and proliferation, as well as ion transport underlie differences in developing *A. aegypti* males vs. females (Tables 1, 2, 3, 4, Figures 2 and 3, Additional files 1 and 3). *In situ* hybridization experiments for a subset of DETs in the pupal brain validated the data set and revealed that the differential expression of a number of the transcripts localized to the optic lobe of the brain (Figures 4 and 11). These results, combined with analysis of *dsx* expression in the developing pupal brain (Figures 8, 9, 10) and the detection of numerous compound eye and eye development GO categories among genes associated with Dsx consensus binding sites (Table 5; Additional file 2), indicate that male and female compound eyes and higher order visual processing may differ in *A. aegypti.* Sex-specific differences in gene expression also localized to the antennal lobe and mushroom body of the 24 h pupal brain (Figures 6 and 7), in which expression of *dsx* was also found to be sexually dimorphic (Figures 9 and 10). These findings suggest that Dsx may contribute to underlying differences in the visual and olfactory systems, the processing of sensory information, as well as learning and memory in *A. aegypti* females and males*.*

As discussed by Wilkinson et al. [72], although sex-biased gene expression has been assessed in several insects, few studies have compared global patterns of gene expression in developing somatic tissue with the goal of understanding a trait that is sexually dimorphic in adults. Their discussion highlights the need for studies like their sex-specific analysis of stalk-eyed fly eye development and our analysis of the *A. aegypti* female vs. male pupal head transcriptome. In addition to performing more studies of this type, it will be critical to functionally assess the roles of the dimorphically expressed genes identified in order to understand if and how these genes contribute to the development of sexually dimorphic traits. This is of course a large task that can be particularly daunting with respect to non-model organisms. However, siRNA-mediated knockdown approaches such as the one employed in this investigation are making this challenging task much more feasible.

## Abbreviations

APF: after puparium formation

*ci*: *cubitus interruptus*

*cdk4/6*: *cyclin-dependent kinase 4/6*

DETs: differentially expressed transcripts

Dsx: Doublesex

DsxF: female-specific Dsx

Fru: Fruitless

GO: gene ontology

GAG: glycosaminoglycan

DsxM: male-specific Doublesex

IDT: Integrated DNA Technology

IGluR: ionotropic glutamate receptor

IR: ionotropic receptor

KD: knockdown

KEGG: Koyota Encyclopedia of Genes and Genomes

LVP-IB12: Liverpool-IB12

*obp10*: *odorant binding protein 10*

Synj: synaptojanin

Wg: Wingless

## Competing interests

The authors declare that they have no competing interests.

## Authors' contributions

MT, KM, PL, SE, DWS, and MDS conceived and designed the experiments. MT, LS, KM, PL, and SE performed the experiments. MT, PL, KM, DWS, and MDS analyzed the data. SE and DWS contributed reagents and analysis tools. MT, KM, SE, DWS, and MDS prepared the manuscript. All authors read and approved the final manuscript.

## Additional files

## Supplementary Material

Additional file 1: **Full list of significant DETs in the male vs. female 24 h pupal head.** Four unique replicates (male 1, 2, 3, 4 and female 1, 2, 3, 4) as well as two repeat replicates (male 5, which is a repeat hybridization of male 1; male 6, a repeat hybridization of male 4; female 5, a repeat hybridization of female 1; female 6, a repeat hybridization of female 2) were assessed. The raw intensity values for each male or female replicate or repeat replicate are indicated. The mean male intensity, mean female intensity, log2 (male/female) fold change, and fold change (male/female) values are indicated for each probe. Raw *p* values adjusted using the Bonferroni procedure for control of false discovery rate are indicated (Bonferroni *p* value) for significant (*p* < 0.05) DETs. File format: Excel spreadsheet (XLSX). Click here for file

Additional file 2: **List of Dsx consensus binding sites identified in the*A. aegypti*genome.** The locations of Dsx consensus binding sites, as well as the genes in which they are contained or flank are indicated. Genes corresponding to significant (*p* < 0.05) DETs in the 24 h female vs. male pupal head microarray data set are noted. File format: Excel spreadsheet (XLSX). Click here for file

Additional file 3: **Genes corresponding to KEGG pathways that are significantly over-represented among DETs significantly upregulated in females or males.** Gene numbers and descriptions for genes corresponding to each significant (*p* < 0.05) KEGG pathway are indicated. File format: Excel spreadsheet (XLSX). Click here for file
